# Study on Fast Liquefaction and Characterization of Produced Polyurethane Foam Materials from Moso Bamboo

**DOI:** 10.3390/ma17153751

**Published:** 2024-07-29

**Authors:** Go Masuda, Satoshi Akuta, Weiqian Wang, Miho Suzuki, Yu Honda, Qingyue Wang

**Affiliations:** 1 Graduate School of Science and Engineering, Saitama University, 255 Shimo-Okubo, Sakura-ku, Saitama 338-8570, Japan; masuda.g.824@ms.saitama-u.ac.jp (G.M.); s.akuta.073@ms.saitama-u.ac.jp (S.A.); weiqian@mail.saitama-u.ac.jp (W.W.); miho@fms.saitama-u.ac.jp (M.S.); 2 LignoMateria Corporation, Akasaka Hukugen Building 1F, Akasaka 2Chome 15-16, Minato, Tokyo 107-0052, Japan; y.honda@lignomateria.co.jp

**Keywords:** fast liquefaction, waste biomass, moso bamboo, polyurethane foam, ethylene carbonate

## Abstract

Although bamboo is widely distributed in Japan, its applications are very limited due to its poor combustion efficiency for fuel. In recent years, the expansion of abandoned bamboo forests has become a social issue. In this research, the possibility of a liquefaction process with fast and efficient liquefaction conditions using moso bamboo as raw material was examined. Adding 20 wt% ethylene carbonates to the conventional polyethylene glycol/glycerol mixed solvent system, the liquefaction time was successfully shortened from 120 to 60 min. At the same time, the amount of sulfuric acid used as a catalyst was reduced from 3 wt% to 2 wt%. Furthermore, polyurethane foam was prepared from the liquefied product under these conditions, and its physical properties were evaluated. In addition, the filler effects of rice husk biochar and moso bamboo fine meals for the polyurethane foams were characterized by using scanning electron microscopy (SEM) and thermogravimetry and differential thermal analysis (TG-DTA), and the water absorption and physical density were measured. As a result, the water absorption rate of bamboo fine meal-added foam and the thermal stability of rice husk biochar-added foam were improved. These results suggested that moso bamboo meals were made more hydrophilic, and the carbon content of rice husk biochar was increased.

## 1. Introduction

**Figure 1:**
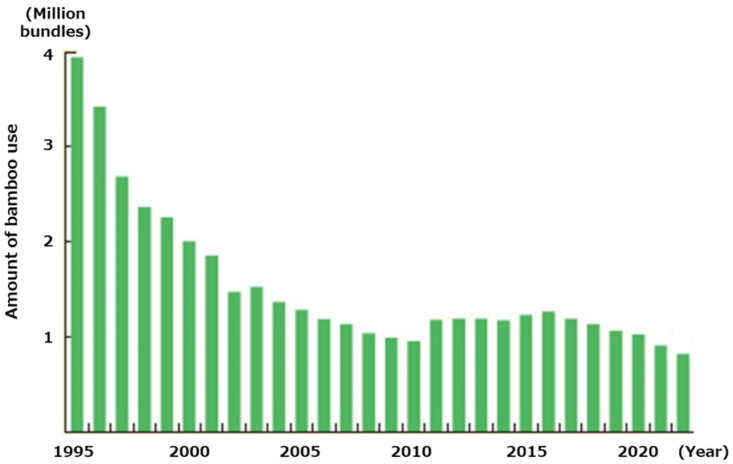
Changes in the amount of bamboo use in Japan [6].

Since the evolution of industry, our society has developed a reliance on fossil fuels. However, in recent years, global warming and climate change have become significant global issues, particularly due to CO_2_ emissions generated from the combustion of plastics or oil in power plants. These emissions are considered the main cause of these issues. Therefore, to reduce the amount of CO_2_ emissions, the utilization of biomass is essential [1]. Biomass resources can capture carbon from the air and provide reusable energy. These resources, which include wood, agricultural waste, and food waste, exist as renewable organic materials. Additionally, utilizing biomass resources is crucial for realizing a sustainable society and a circular economy by maximizing the value of resources and minimizing irrecoverable waste [2]. However, biomass is less processable compared to conventional plastics. For many years, large amounts of biomass resources have gone unused, except for burning or landfilling, which generates severe pollution problems [3,4,5]. This indicates that there are still few applications for biomass materials currently. In this study, one type of unused biomass, moso bamboo, was chosen as the raw material to conduct the research [6]. The bamboo samples in this study were bamboo that was cut down and left abandoned in the forest. Bamboo nowadays has less value because it has poor combustion efficiency and its physical strength is lower than that of wood, making it difficult to use as a building material. For these reasons, a large amount of unused bamboo has been left abandoned, and this research aims to make effective use of it in a different way than before. Moso bamboo is widely distributed in Asian countries. Due to its rapid growth rate, it has been reported that if bamboo forests are abandoned, they will naturally expand quickly, encroaching on other forests and agricultural fields and becoming a social issue. Additionally, there are few applications for bamboo [6]. Thus, once bamboo forests are abandoned, they expand rapidly [6]. In Japan, the utilization of bamboo is decreasing while the area of bamboo forests, including abandoned ones, is increasing, as shown in Figure 1. This highlights the importance of developing new applications for this biomass resource.

Liquefaction technology has been identified as a reasonable method to convert biomass to bio polyols, which can be used to synthesize bioplastics as raw materials. Extensive research has been conducted in this field, exploring the thermal liquefaction of various unused biomass materials under different temperatures and solvent systems [7,8,9,10]. In this research, it was found that adding glycerol to prevent recondensation, and ethylene carbonate to boost the liquefaction speed, provided more suitable conditions [11]. Especially, ethylene carbonate accelerates the dissolution of cellulose because of its high permittivity. By using HPLC and ^13^C-NMR, Yamada et al. suggested the mechanism for the dissolution of cellulose [12]. This faster liquefaction process enables energy savings and increases production speed, which are crucial for industry when utilizing biomass resources. While the petrochemical industry has optimized production over decades, the biomass industry still needs to enhance its efforts to achieve efficient mass production. Therefore, this study focused on achieving more efficient and rapid liquefaction with a low environmental impact. Typically, the liquefaction process requires an acid catalyst, such as sulfonic acid, with the amount depending on the liquefaction temperature and solvent volume. To achieve high efficiency and low environmental impact, a polyethylene glycol/glycerol mixture solvent system with ethylene carbonate was selected. The study optimized the liquefaction process with different ethylene carbonate contents and varying catalyst amounts.

In a second part, a liquefied product derived from moso bamboo was used for making polyurethane foam (PUF). PUF is widely used for cushioning material, for thermal isolation, and as an agricultural substrate, etc. To increase biomass usage, a biobased cyanate resin, STABiO, was selected for this research. According to Tuyet et al. [13], loading the nano-silica particles derived from rice husk could enhance its thermal stability and density. Dorota et al. [14] reported that sunflower shells, rice husks, and buckwheat husks also provide similar improvements. Thus, in this study, two different fillers, bamboo meal filler and rice husk biochar, were selected, and a PUF with these fillers was made. The two different fillers were selected with the expectation that bamboo meal would increase the biomass content, and rice husk biochar the thermal stability. Furthermore, we executed not only the production of foam but also examined characteristics such as water adsorption and thermal properties compared to foam without any fillers.

## 2. Materials and Methods

### 2.1. Materials

Waste moso bamboo was collected from the bamboo forest in Inagi city, Tokyo. Collected moso bamboo consisted of culm and branch parts. Collected moso bamboos were cut down and stored for around 4 months in the bamboo forest. All parts of the bamboo except for the leaves were used for the liquefaction process. Rice husk was provided by a local rice famer near the University of Saitama.

For the liquefaction process, polyethylene glycol 400 (PEG400), glycerol, ethylene carbonate, and sulfonic acid were purchased from Wako Pure Chemicals, Ind. Ltd., Osaka, Japan. Additives used in the polyurethane foaming process included the cyanate resin, STABiO (Mitsui Chemicals, Tokyo, Japan), silicone surfactant (SH193; Toray Dow Corning Silicone, Ind. Ltd., Tokyo, Japan), and dibutyltin dilaurate (Wako Pure Chemicals, Ind. Ltd., Osaka, Japan). All other reagents were purchased as analytical grade (Wako Pure Chemicals, Ind. Ltd., Osaka, Japan) and used without further purification.

### 2.2. Preparation of Bamboo Meal

To obtain bamboo meal samples between 100 to 500 μm, the following procedure was carried out. All bamboo was dried sufficiently under 1 atm, and the moisture of the bamboo sample was 5.7 wt%. The original bamboos were cut using a saw and then ground with a blender (WB-1 700 W OSAKA CHEMICAL, Osaka, Japan). Fine bamboo meal samples 100 to 500 μm in size were obtained using an impact grinder (MF 10.2 IKA, Staufen, Germany). Then, the bamboo meal samples were dried in an oven at 105 °C for 1 h before use. At 110 °C, the samples were then defatted through Soxhlet extraction for 6 h. A total of 50 mL of ethanol and 100 mL of benzene were used for the defatting process. After the 6 h Soxhlet extraction, the sample was vacuum-filtered and thoroughly dried in an oven.

### 2.3. Estimating the Content of Lignin and Holocellulose (Klason Lignin and Wise Method)

The content of holocellulose and lignin was measured following the same method as in a previous study [15]. Analysis of the content ratio of lignin was performed by the Klason lignin method. First, 1.0 g of the bamboo sample and 15 mL of 72% sulfuric acid were put in a 50 mL beaker and stirred occasionally. After four hours, these contents were poured into a 1 L flask, which was filled with 560 mL of extra-pure water. This flask was fitted with a condenser and heated at 110 °C in an oil bath for four hours. These contents were then filtered using a glass filter (1GP16, AS ONE Co. Ltd., Amagasaki, Japan) and rinsed with hot water and pure water. The residual solid left was lignin.

Analysis of the content ratio of holocellulose was performed by the Wise method. First, 2.5 g of the bamboo samples was put in a 300 mL flask with 150 mL of pure water, 1.0 g of sodium chlorite, and 0.20 mL of acetic acid, and then heated in a water bath at 75 °C with occasional stirring; 1.0 g of sodium chlorite and 0.2 mL of acetic acid were added three times at four-hour intervals. Then, these contents were filtered using a glass filter (1GP100, AS ONE Co. Ltd., Japan) and rinsed with pure water and acetone. The residual solid was holocellulose.

### 2.4. Elemental Analysis of Bamboo Using CHN Analyzer

To analyze the organic components of moso bamboo, we measured the components using an Organic Micro Element Analyzer (CHN analyzer; MT-5 Yanaco Co. Ltd., Tokyo, Japan), with temperatures set at 950 °C for Combustion Furnace, 850 °C for Oxidation Furnace, and 550 °C for Reduction Furnace, using helium as the carrier gas. In this study, antipyrine was used as the standard sample [16].

### 2.5. Liquefaction of Bamboo

**Figure 2:**
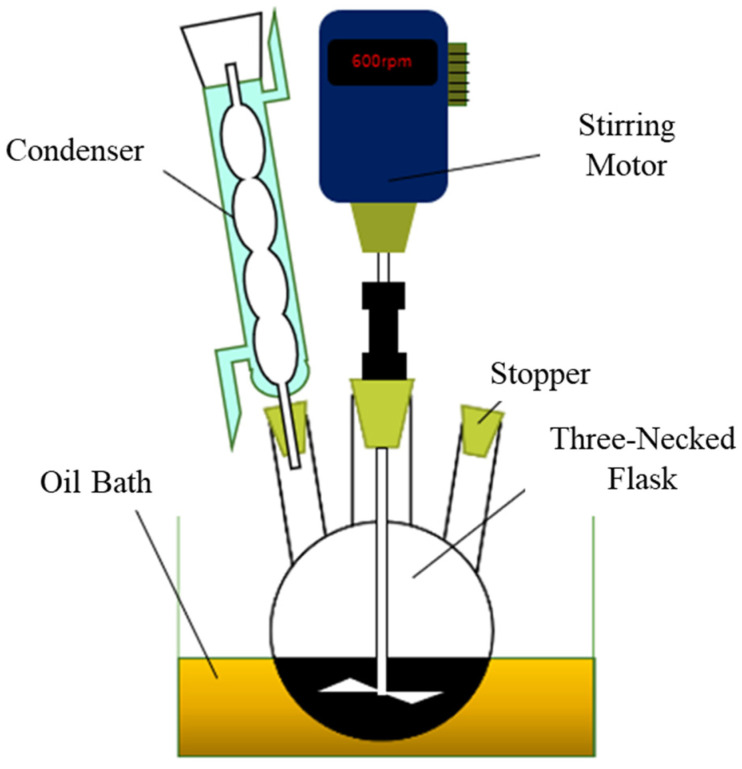
Liquefaction equipment settings.

As shown in Figure 2, 10 g of bamboo meal and 40 g of the mixed solvent were added to a 500 mL three-neck reaction flask equipped with a stirrer and a reflux condenser, as specified in Table 1. The reaction flask was then placed in a 150 °C heated oil bath. The reaction was started by adding the catalyst (H_2_SO_4_) to the reaction flask. After liquefaction times of 15, 30, 60, 90, and 180 min, the liquefied products were collected and placed in a weighted vial for residue content analysis. To stop the liquefaction reaction, the vial was quenched in cold water. After stopping the reaction, methanol was added to dissolve the liquefied product, and then the liquefied product’s pH was adjusted to 7.0 with magnesium oxides. Filtering to remove all salts and evaporation of all methanol were carried out. Following these processes, the liquefied products derived from moso bamboo were obtained. All liquefaction applied in this research are shown in Table 1.

### 2.6. Characterization of the Liquefied Bamboo

To evaluate liquefaction, the functional groups of the liquefied bamboo samples were characterized through Fourier-transform infrared spectroscopy (FT-IR) (JASCO Co. Ltd., Tokyo, Japan). Thinly clipped liquefied bamboo samples were positioned between potassium bromide plates, and the resultant pellet was inserted into the FT-IR apparatus to determine the functional groups [17].

For residue quantification, 10 mL of methanol was introduced into the vial and thoroughly mixed. Filtration was executed using a vacuum pump and paper filters (ADVANTEC C5, Toyo Roshi Kaisha, Ltd., Tokyo, Japan). The filtered residue was subsequently dried for 24 h in an oven set at 105 °C. The residue percentage was determined using Equation (1).

(1) Ratio of residue (%) =W1/W2 × 100

where W1 is the weight ratio of residue in the taken liquefied sample; and W0 is the weight ratio of bamboo in the initial sample.

The average molecular weights of liquefied bamboo samples were measured using a gel permeation chromatography (GPC) system equipped with a column (KF-802, Shodex, Co. Ltd., Yokohama, Japan), an HPLC pump (Pump PU-2080, JASCO Co. Ltd., Japan), a column thermostat (CO-2060, JASCO Co. Ltd., Tokyo, Japan), and a refractive index-detector (RID) (RI-2031, JASCO Co. Ltd., Tokyo, Japan). Following our previous research, the analytical conditions are shown in Table 2 [18]. Exactly 10 mg of the liquefied bamboo samples was diluted with 2.5 mL of tetrahydrofuran (THF) and then analyzed by GPC.

The hydroxyl value of the liquefied wood is an important factor as it determines the mechanical properties of the prepared resin. The acid number and hydroxyl values of liquefied wood were measured and adapted from Ertas et al. [19]. The hydroxyl value was calculated with acid values. The hydroxyl and acid values of liquefaction wood were determined by titration. Blank titration was conducted using the same procedure.

### 2.7. Preparation of Fillers for the Polyurethane Foams (PUF)

Bamboo meal with a particle size of under 100 μm was used as filler as-is, without any further process. The other filler was derived from rice husk collected from the vicinity of Saitama University. The preparation of rice husk biochar followed this procedure: A ceramic cup containing rice husks was placed in a muffle furnace and heated to 900 °C to incinerate all organic compounds. After cooling to room temperature, the residues were ground using a mortar. An electromagnetic sieve shaker was employed to isolate particles smaller than 100 μm.

The rate of filler loading in PUF was selected as 7, 13, and 18 wt% vs. total weight. In addition, the residue of the liquefied product was also investigated in the same way as bamboo fillers.

### 2.8. Preparation of Polyurethane Foam (PUF)

Table 3 presents the conditions for polyurethane foam (PUF) production utilized in this study. The sample names include the weight in grams, which represents the amount of material used for foam preparation. The liquefied product was synthesized using a mixed solvent comprising polyethylene glycol 400 and glycerol in a 70/30 ratio, with the addition of ethylene carbonate constituting 20 wt% of the total solvent weight. The [NCO]/[OH] ratio denotes the mixture proportions of isocyanate and hydroxyl functional groups. The [NCO] value was obtained from the supplier’s specifications, while the [OH] value was determined through measurement and calculation based on the previously described acid values. A ratio of 1 signifies an equimolar presence in these functional groups.

Liquefied bamboo samples were combined with ultra-pure water and a silicone surfactant in a paper cup, and the mixture was stirred at 2000 rpm for 2 min. Subsequently, biobased cyanate resin (STABiO D-370N) and a curing catalyst, dibutyltin dilaurate (DBTDL), were added, and the mixture was further stirred at 3000 rpm for an additional 2 min. To cure the resin, the samples were left to stand at room temperature for over 24 h. Following the curing process, the samples were extracted from the cup, and their properties were subsequently evaluated.

### 2.9. Scanning Electron Microscopy (SEM) of PUF

To investigate the cell size and morphology of the polyurethane foam (PUF), scanning electron microscopy (SEM) was conducted using a S-2400 instrument (HITACHI High-Tech, Tokyo, Japan). The samples were sectioned to approximately 1 mm thickness with a knife. The images were obtained following osmium sputter coating to prevent electrical charging during SEM analysis. The SEM was operated at an accelerating voltage of 15 kV, and the images were observed at a magnification of 100×.

### 2.10. Measurement of Water Absorption Rate of PUF

A water absorption test was conducted according to Japanese Industrial Standards K7209 [20]. Sample weights were measured precisely using an electric balance, and the water absorption rate was calculated using Formula (2), shown below.

(2) $$Water\;absorption\;rate\;\left(\%\right)=\frac{\left(m_{2}-m_{3}\right)}{m_{1}}\times 100\;$$

*m*_1_: the sample weight before water soaking (mg)
*m*_2_: the sample weight after water soaking (mg)
*m*_3_: the sample weight after drying (mg)

### 2.11. Thermal Decomposition

To evaluate the thermal decomposition characteristics of polyurethane foams, simultaneous thermogravimetry and differential thermal analysis (TG-DTA) were performed using a TG-DTA device (DTG-60, Shimadzu Corporation, Kyoto, Japan). The thermal behavior of polyurethane foam is important because foam is mainly used as a thermal insulator. The measurements were carried out with a ramp speed of 10 °C·min^−1^ in ambient air [21].

## 3. Results and Discussion

### 3.1. Componential Analysis of Moso Bamboo

The results of the componential analysis were as follows: carbon (C) 47%, hydrogen (H) 6%, nitrogen (N) 0.3%, and oxygen (O) 46%. The ash content was 1.0%, which is relatively low compared to other studies [22]. Ash content varies significantly depending on the soil in which the bamboo is grown. These results are presented in Table 4 below.

Using the Wise method and Klason lignin analysis, the amounts of holocellulose and lignin were determined to be 74% and 28%, respectively. The lignin content in biomass depends on the kind of plant, with bamboo typically containing around 30% lignin. Lignin and hemicellulose are firstly liquefied in the process [12], so around 30% lignin content is expected to be efficient for liquefaction. Thus, the samples were concluded to have enough lignin content for the liquefaction process. The results are shown in Table 5.

**Figure 3:**
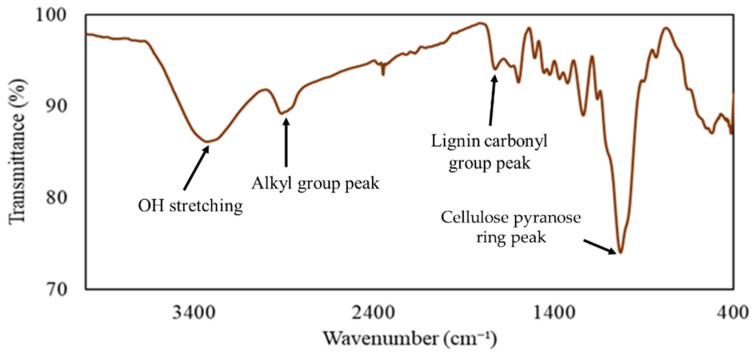
FT-IR spectrum of moso bamboo meals.

To check the availability of the functional groups of moso bamboos, FT-IR was carried out, and the spectrum is shown in Figure 3. An OH stretching vibration peak at around 3300 cm^−1^, an alkyl group peak at around 2890 cm^−1^, a lignin carbonyl group peak at around 1725 cm^−1^, and a cellulose pyranose ring peak at around 1022 cm^−1^ were observed [23,24]. These results also suggested that unused bamboo contains enough biomass compounds and functional groups for the liquefaction process. Thus, this bamboo meal was used in the next step to make the liquefied product.

### 3.2. Evaluation of Liquefied Products

#### 3.2.1. Optimization of the Bamboo Liquefaction Process

**Figure 4:**
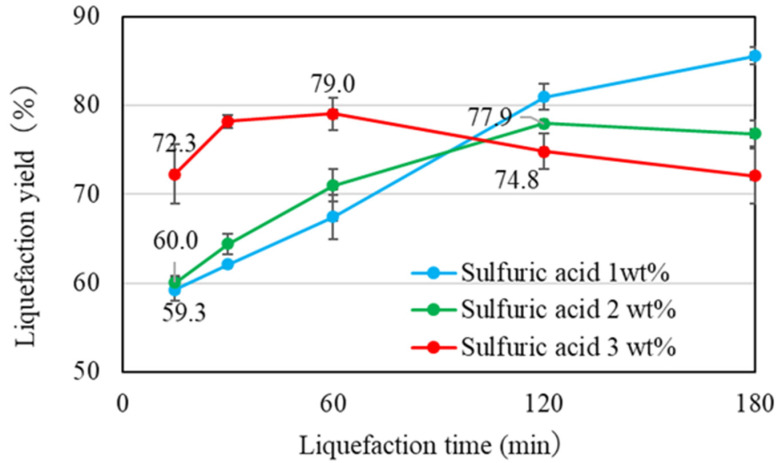
Comparison of residue content by amount of sulfuric acid.

In this study, optimization of the liquefaction process efficiency was achieved by analyzing the residue content [25]. The optimization parameters included reaction time, the quantity of sulfuric acid, and the composition ratio of ethylene carbonate. Figure 4 shows the variation in residue content as a function of the amount of sulfuric acid. The mixed solvent used for the liquefaction process consisted of polyethylene glycol 400 and glycerol in a 70/30 ratio.

By increasing the amount of sulfuric acid from 1 wt% to 3 wt%, the liquefaction yield was also increased. The liquefaction yield of 3 wt%, however, decreased after a 60 min liquefaction time. This suggested that after the 60 min liquefaction time, recondensation between lignin and cellulose derivatives occurred [26]. Despite the liquefaction yield of 1 wt% being further increased, the liquefaction yield of 2 wt% became stable after 120 min. This suggested that a higher sulfuric acid concentration led to the requirement for a shorter reaction time to achieve the maximum liquefaction yield. In this study, the target liquefaction yield was set to around 80% from this experiment. This rate could be obtained using 3 wt% sulfuric acid with a 60 min liquefaction or 2 wt% sulfuric acid with a 120 min liquefaction.

**Figure 5:**
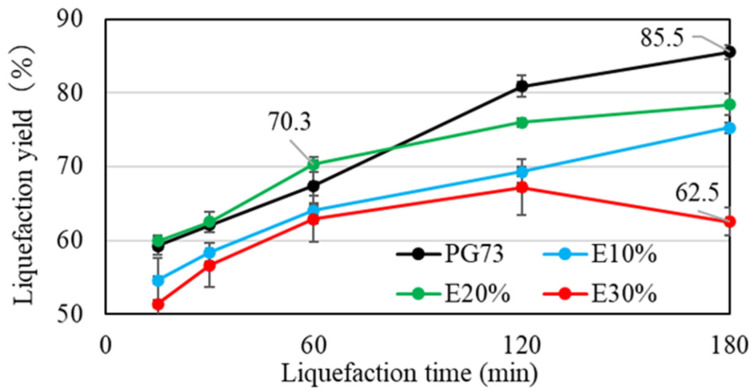
Comparison of residue content among different amounts of ethylene carbonate with 1 wt% sulfuric acid.

**Figure 6:**
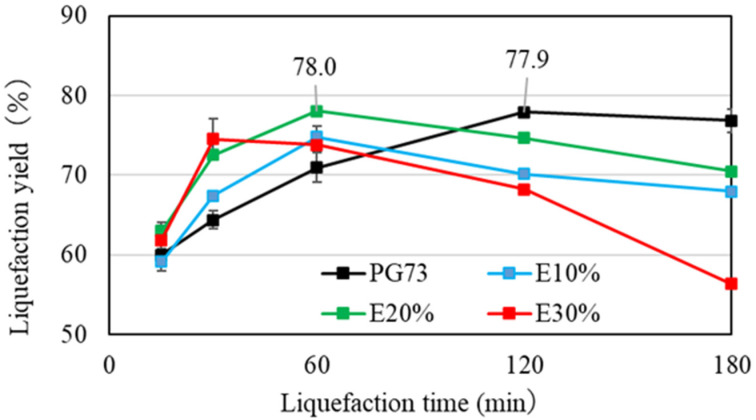
Comparison of residue content among different amounts of ethylene carbonate with 2 wt% sulfuric acid.

To accelerate the liquefaction speed and efficiency, ethylene carbonate was added to the liquefaction process [27,28,29]. If ethylene carbonate can boost the process efficiency, a similar liquefaction yield can be obtained with reactions with less sulfuric acid, and within a short reaction period. Thus, catalyst reactions with 1% and 2% acid were focused on. Polyethylene glycol 400/glycerol (70/30) was used as the mixed solvent. E10 means that ethylene carbonate 10 wt% was added to the total mixed solvent. PG73 means that the reaction was performed without ethylene carbonate. The results are shown in Figure 5 and Figure 6.

In the case of 1% sulfuric acid, it took more than 180 min to reach an 80% liquefaction yield, regardless of the amount of ethylene carbonate added. This indicates that efficiency could not be improved with 1% sulfuric acid, even with the addition of ethylene carbonate. Thus, adding ethylene carbonate in this scenario was pointless. In contrast, in the case of 2% sulfuric acid, with 20% ethylene carbonate, the liquefaction yield became 78% after 60 min, almost reaching the target value of 80%. This condition made it possible to shorten the reaction time from 120 min to 60 min. In the case of 30% ethylene carbonate, however, a decreasing liquefaction yield was observed before the liquefaction yield reached 80%. This was because adding a large amount of ethylene carbonate also increased the recondensation reaction. A similar trend was observed in the case of 1% sulfuric acid.

#### 3.2.2. Measurement of the Molecular Weight of the Liquefied Products with Gel Permeation Chromatography (GPC)

**Figure 7:**
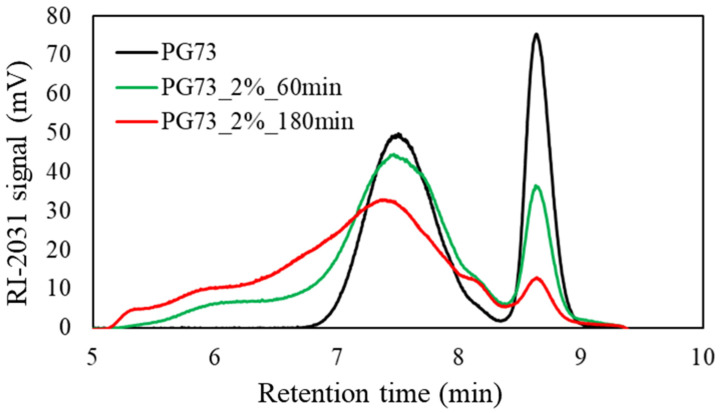
GPC chromatography of the liquefied products without ethylene carbonate.

**Figure 8:**
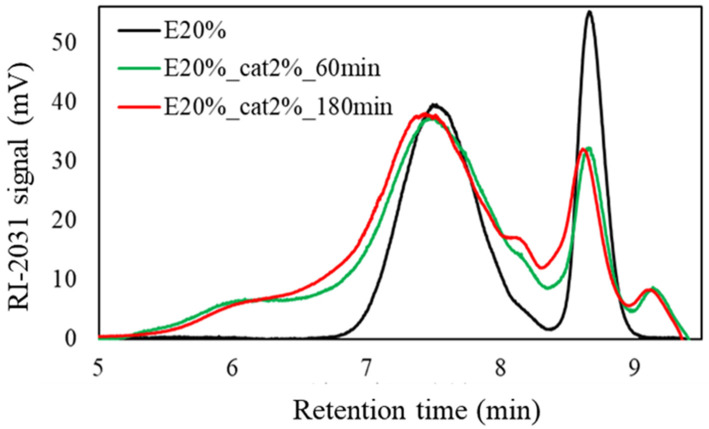
GPC chromatography of the liquefied products with ethylene carbonate.

GPC measurements were performed to compare the molecular weight of the liquefied products with and without ethylene carbonate depending on the liquefaction time. The amount of sulfuric acid was 2%, and the solvent used was polyethylene glycol 400/glycerol (70/30). The results are shown in Figure 7 without ethylene carbonate and in Figure 8 with ethylene carbonate (20 wt% of the total solvent weight). The molecular weight (Mw), molecular number (Mn), and molecular weight dispersion of these measurements are shown in Table 6.

Under liquefaction conditions without ethylene carbonate, the glycerol peak (retention time 8.6 min) became smaller with the liquefaction time, and the polyethylene glycol 400 peak (retention time 7.5 min) was broader. This phenomenon indicated that liquefaction proceeded while the solvent was consumed. In addition, molecular weight dispersion (Mw/Mn) was increased with the liquefaction time. This suggested that a recondensation reaction was occurring.

On the other hand, in the case of liquefaction with the addition of ethylene carbonate, there was no significant difference between the liquefaction times of 60 and 180 min. During this period, the liquefaction yield was also stable. This result suggested that the liquefaction reaction was completed at 60 min. At the same time, molecular weight dispersion (Mw/Mn) did not increase even at 180 min, suggesting that the recondensation reaction did not occur.

#### 3.2.3. Evaluation of the Functional Groups of the Liquefied Products with FT-IR

**Figure 9:**
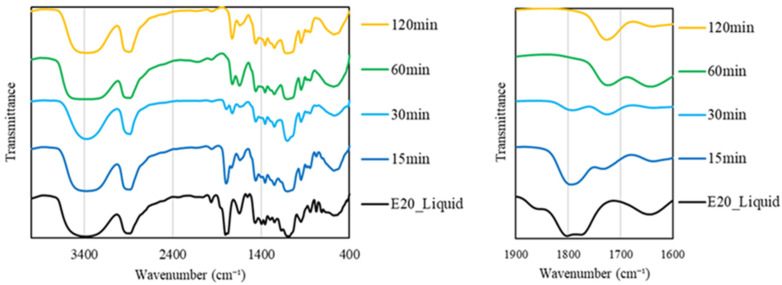
FT-IR spectra of the liquefied products with ethylene carbonate.

The FT-IR spectrum of the change in liquefaction time in a solvent with 2% sulfuric acid and 20% ethylene carbonate, which resulted in a liquefaction yield of 78.0%, is shown in Figure 9. The right side of the figure shows an enlarged view of the wavelengths between 1900 cm^−1^ and 1600 cm^−1^.

The functional group structure in the liquefied product was considered based on the change in the carbonyl group peak and the ethylene carbonate peak.

Up to the 30 min reaction time, a high-frequency C=O stretching vibration peak at 1800 cm^−1^ derived from ethylene carbonate was confirmed. On the other hand, after 60 min, this peak completely disappeared, and a peak around 1730 cm^−1^ that could not be confirmed in the solvent appeared. This peak around 1730 cm^−1^ is considered to be the C=O stretching vibration of aldehydes, ketones, esters, and carboxylic acids derived from the components in the moso bamboo by liquefaction. Therefore, it is thought that the ethylene carbonate added to promote the liquefaction reaction was completely decomposed and became part of the liquefied product with hydroxyl groups via ethylene glycol.

### 3.3. Evaluation of Polyurethane Foam Derived from Liquefied Products

#### 3.3.1. Evaluating the Functional Groups of Polyurethane Foams with FT-IR

**Figure 10:**
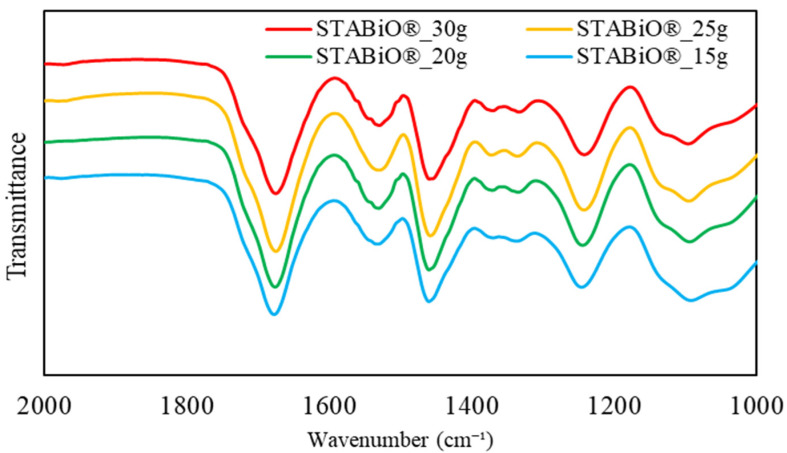
FT-IR spectra of polyurethane foams with different amounts of STABiO.

The FT-IR spectra of polyurethane foams made by changing the ratio of cyanate resin are shown in Figure 10. The numbers written after STABiO indicate the weight added, with the cyanate/polyol ratios of STABiO^®^_15 g to 30 g meaning 0.6, 0.8, 1, and 1.2, respectively.

**Figure 11:**
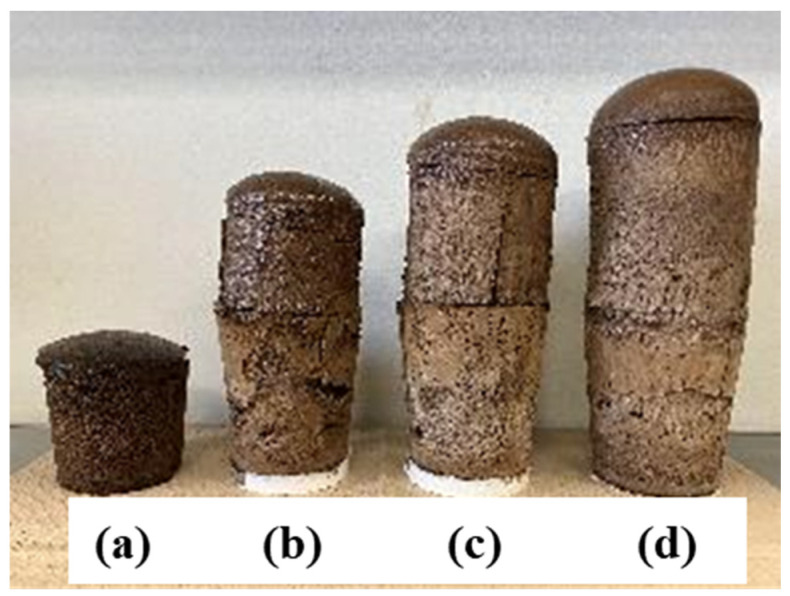
Appearance of polyurethane foams of different cyanate/polyol ratios after foaming: (**a**) 0.6, (**b**) 0.8, (**c**) 1, (**d**) 1.2.

From this spectrum, no significant difference was observed in the spectra for any of the cyanate/polyol ratios. This suggested that there was no difference in the functional groups contained, including urethane bonds. On the other hand, as shown in Figure 11, there was a large difference in the foaming ratio.

The amount of foaming increased with the increase in the ratio of isocyanate/polyol from (a) to (d). Furthermore, in the samples with cyanate/polyol ratios of 0.6 and 0.8, an unreacted part was found inside the foam even after the curing reaction. On the other hand, no unreacted part was found in samples with a cyanate/polyol ratio of 1 or more. As a result, at least the ratio of cyanate/polyol should be 1 or more.

#### 3.3.2. Morphology of Polyurethane Foams with Scanning Electron Microscopy (SEM)

**Figure 12:**
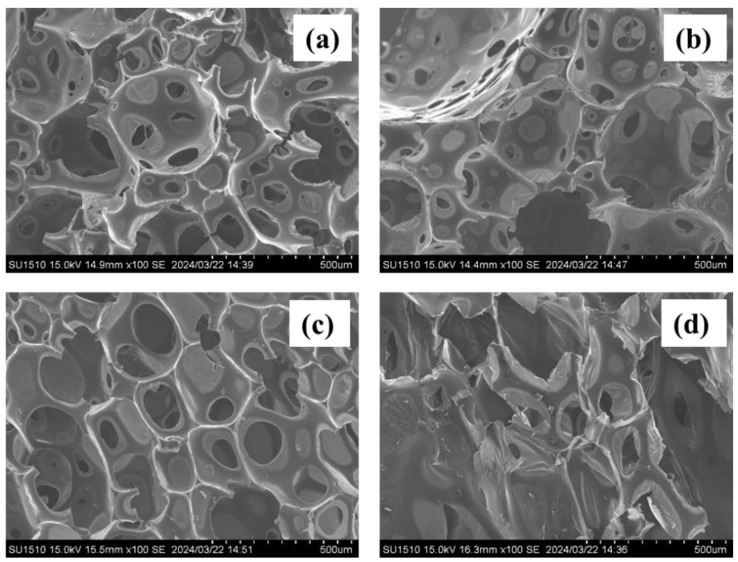
SEM images of polyurethane foams with different cyanate/polyol ratios. (**a**) 0.6, (**b**) 0.8, (**c**) 1, (**d**) 1.2.

To evaluate the morphology of polyurethane foams with liquefied products, scanning electron microscopy (SEM) was performed. The SEM images of the polyurethane foams are shown in Figure 12.

There was a tendency for the smaller distances between cells to decrease as the cyanate/polyol ratio increased. This is considered to improve the strength and thermal insulation properties of the foam. In addition, as the cyanate/polyol ratio increased, the foaming became excessive, and the specific gravity decreased in proportion to the amount of foam.

However, in (d), where the cyanate ratio was 1.2 times that of the polyol, the cell structure could not be maintained uniformly, and the cells collapsed. As this cannot be said to be optimal in terms of strength and thermal insulation, (c), with a cyanate/polyol ratio of 1/1, was considered as the optimal amount to add this time.

#### 3.3.3. Thermal Properties of Polyurethane Foam with Fillers

To improve the thermal stability of polyurethane foams with liquefied products, adding some fillers such as extra bamboo meals and rice husk biochar was considered. In addition, the residue of liquefied product (with a residue of LW) was used as the filler.

**Figure 13:**
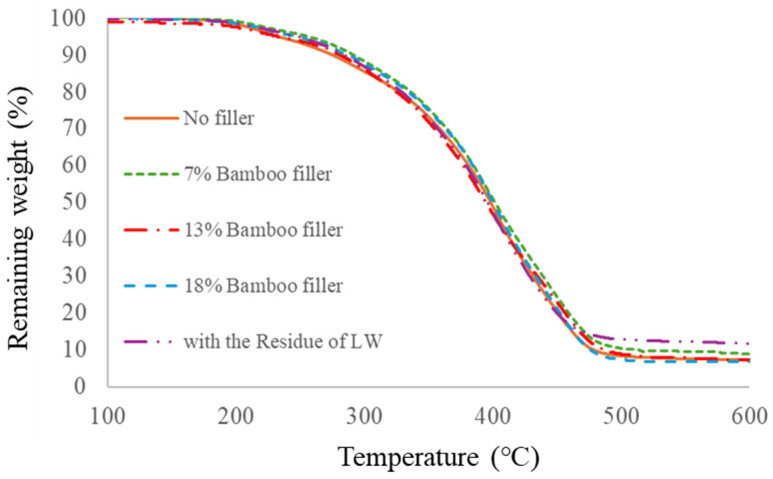
TG-DTA data of polyurethane foams with bamboo meals.

Figure 13 and Table 7 show the TG-DTA data of polyurethane foams with different bamboo meal fillers loaded.

Foams with filler loading showed slightly higher thermal stability than the no-filler foam. The temperature for 50% weight loss, however, seemed almost equal to that for the no-filler foam. This indicated that the bamboo meal filler was an organic compound, so it did not increase the thermal stability of the foams as drastically. In addition, the residue of the liquefied product seemed to work as the organic filler according to these data.

**Figure 14:**
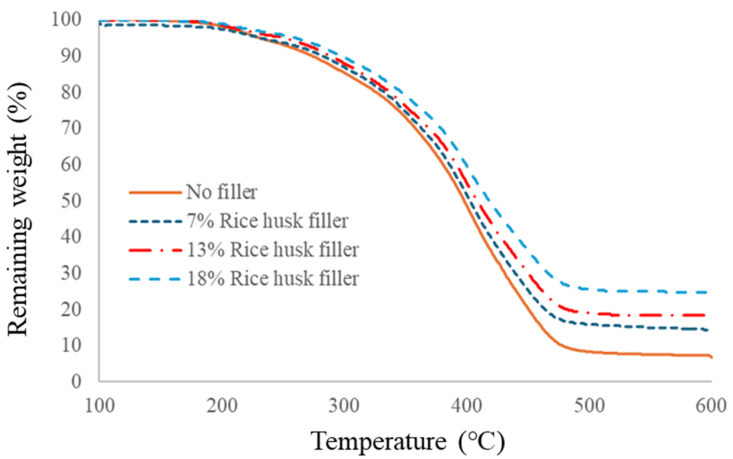
TG-DTA data of polyurethane foams with rice husk filler.

Figure 14 and Table 8 show the TG-DTA data of polyurethane foams with different rice husk fillers loaded.

In contrast to the bamboo meal filler, the rice husk filler seemed to improve the polyurethane foam’s thermal stability. With increasing amounts of the rice husk filler, the temperature of the weight loss was increased as well. In this case, the rice husk filler was inorganic, so this inorganic filler increased the thermal stability of the polyurethane foams.

#### 3.3.4. Morphology of Polyurethane Foams with Fillers Shown by Scanning Electron Microscopy (SEM)

**Figure 15:**
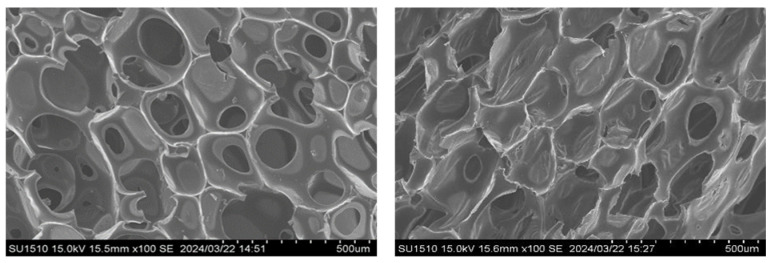
SEM images of polyurethane foams (**left**) without residue and (**right**) with residue.

**Figure 16:**
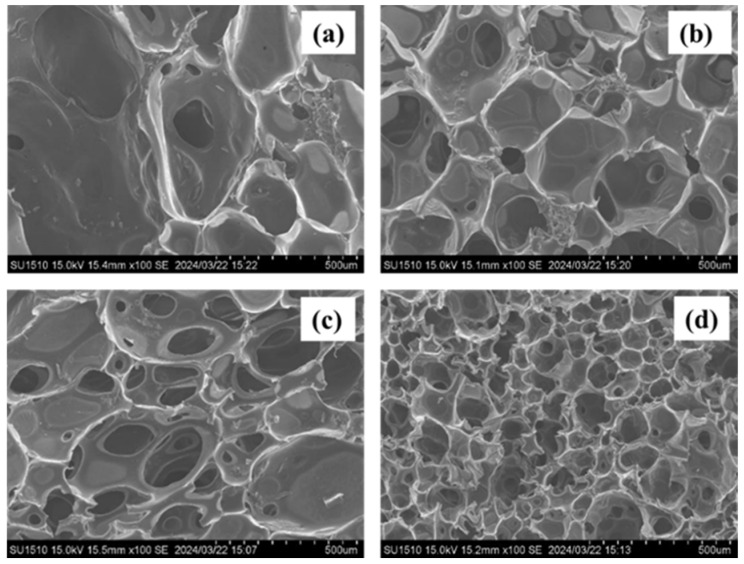
SEM images of polyurethane foams with different fillers: (**a**) foam with 7% bamboo filler, (**b**) foam with 18% bamboo filler, (**c**) foam with 7% rice husk filler, (**d**) foam with 18% rice husk filler.

To evaluate the morphology of the foam’s cell size and the effect of the fillers, scanning electron microscopy (SEM) was performed. The SEM images of foams with or without a residue of the liquefied product are shown in Figure 15, and foams with bamboo fillers and rice husk fillers are shown in Figure 16.

From the SEM images in Figure 16, the foam cells seemed to be slightly smaller and deformed, but no significant differences were observed. The difference in cell size may affect the hydrophobic property or change the surface tension of the liquefied product. In addition, the heat resistance slightly improved, and if there was no change in the foam state, it was preferable to make a foam using the liquefied product with residue.

It was observed that the foam cells became smaller as the amount of filler added increased in the case of both fillers. However, looking at the difference between these two fillers, the bamboo meal was more like aggregates. The rice husk biochar was small in particle size, which affected the surface tension of the liquefied product, causing the cell size to become smaller during foaming.

**Figure 17:**
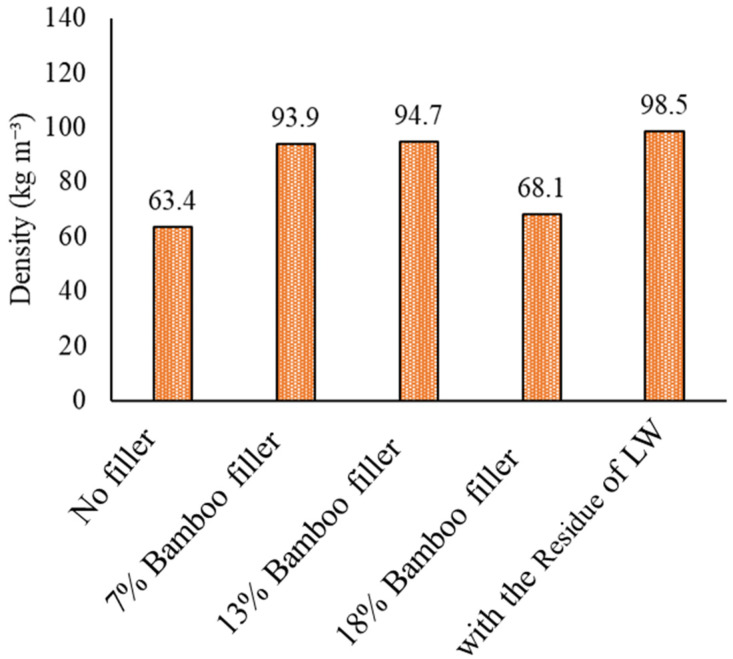
The foam density with bamboo filler.

**Figure 18:**
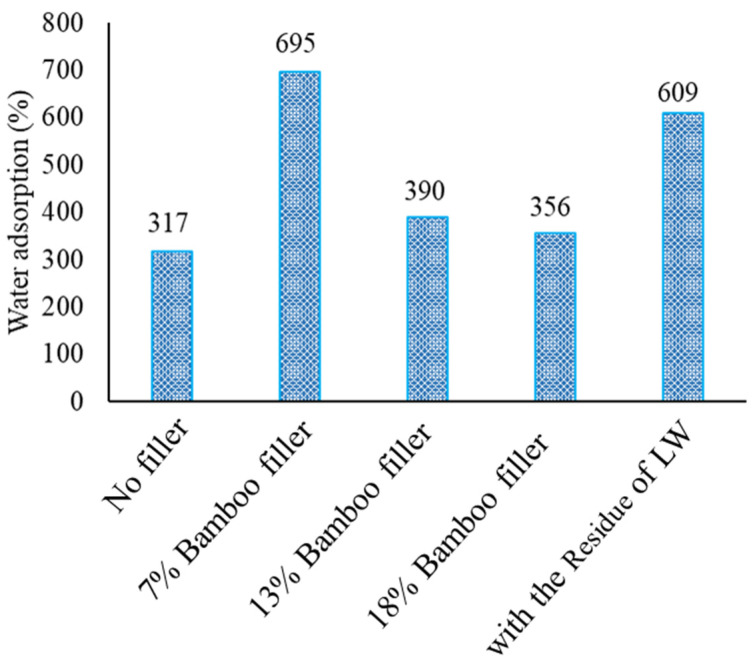
The foam water absorption with bamboo filler.

Figure 17 and Figure 18 show the foam density and the amount of water absorption with bamboo filler and the residue of the liquefied product, respectively.

The foam density increased when the bamboo filler was loaded. The foam density, however, dropped for the 18% loading sample. This was because the foaming ratio was too high, and the decrease in density was greater than the effect of adding the filler. As for the water absorption, it was approximately twice as high at 7% loading compared to the value with no filler. This is presumed to be due to the bamboo meal itself absorbing water. At loading rates higher than 7%, however, the water absorption decreased. This was considered to be due to the cell size being made smaller and increasing the number of closed cells, which made the water absorption rate lower. Therefore, in this study, a large change in water absorption was obtained with 7% bamboo meal loading. This result indicated that with a small amount of bamboo, the filler could sufficiently increase the water absorption rate of a foam.

With the liquefaction process, the possibility of utilizing bamboo as a raw material was proven. It is hoped that liquefying fallen and abandoned bamboo and making polyurethane foam in this way will serve as a new and unprecedented way of using bamboo effectively.

## 4. Conclusions

In this study, ecologically efficient liquefaction conditions for moso bamboo, which has not been used often, were investigated. It was found that adding 20 wt% ethylene carbonate to the polyethylene glycol/glycerol mixed solvent system and reducing the amount of sulfuric acid catalyst from 3 wt% to 2 wt% provided optimal conditions. As a result, this process achieved a liquefaction yield of 78% while maintaining a low environmental impact. Additionally, these conditions successfully shortened the liquefaction time by 50%, reducing it from 120 min to 60 min, thereby also saving energy. Then, polyurethane foam was prepared using this liquefied product as a polyol, and the physical properties were evaluated by changing the cyanate ratio. A higher cyanate ratio increases the foaming ratio and improves weight loss. The SEM observation results showed that the cyanate ratio of 1 provided uniform cell sizes, and it was concluded that this was the optimized state as a foam.

In addition, to further improve the physical properties, rice husk biochar (inorganic) and bamboo meals (organic) were added as fillers, and the physical properties were compared and evaluated with those without fillers. Examining fillers with 7 to 18 wt% loading showed that the thermal properties were improved with rice husk biochar (inorganic), and the water absorption rate was improved with bamboo meals (organic). In particular, the water absorption improvement is due to the difference in the cell size and the number of through holes. In addition, it is considered that polyurethane foam with excessive bamboo meal loading had cells that were too small, resulting in a decrease in water absorption. It was observed that polyurethane foams with liquefied product residue led to an increased density and water adsorption rate of the foams. These results suggested that the residue compounds, while they could not be seen in SEM images, made the foam cell walls more hydrophilic. This means that the addition of the residue compounds itself led to the improved surface hydrophilic properties of the foam.

## Figures and Tables

**Table 1:** Liquefaction conditions.

Mixed solvent	Polyethylene glycol 400/glycerol = 7:3 by weight
Catalyst ratio	Bamboo meal: catalyst = 1, 2, 3% by weight ratio
Added ethylene carbonate ratio	10, 20, 30% by weight rate of mixed solvent

**Table 3:** Polyurethane foam production conditions.

Sample Name	Mixture Ratio	Isocyanate	Liquefied Product	DBTDL	Silicone Surfactant	Water
	[NCO]/[OH]	[g]	[g]	[g]	[g]	[g]
STABiO^®^_15 g	0.6	15	15	1.0	1.0	1.0
STABiO^®^_20 g	0.8	20	15	1.0	1.0	1.0
STABiO^®^_25 g	1	25	15	1.0	1.0	1.0
STABiO^®^_30 g	1.2	30	15	1.0	1.0	1.0

**Table 2:** The analytical conditions of GPC.

GPC	Conditions
Mobile phase	Tetrahydrofuran
Flow rate	1 mL/min
Dilute concentration of sample	0.4 g L^−1^
Tetrahydrofuran column temp.	40 °C
Volume of sample loop	100 μL

**Table 4:** The componential analysis of moso bamboo.

Particle Size	C (%)	H (%)	N (%)	O (%)	Ash (%)
100–500 μm	47	6.0	0.3	46	1.0

**Table 5:** The results of Wise and Klason lignin methods.

Particle Size	Klason Lignin (%)	Holocellulose (%)	Organic Volatile (%)
100–500 μm	28	74	1.4

**Table 6:** Molecular weight/number of GPC measurements without ethylene carbonate (left) and with ethylene carbonate (right).

Time	Mn	Mw	Mw/Mn	Time	Mn	Mw	Mw/Mn
0	303	340	1.12	0	307	391	1.27
60	375	585	1.56	60	355	586	1.65
180	477	946	1.98	180	358	551	1.54

**Table 7:** Weight loss data of polyurethane foams with bamboo meals.

Sample	5% Weight Loss	10% Weight Loss	Max DTG Point	50% Weight Loss
No filler	237	276	390	395
7% bamboo filler	256	291	389	402
13% bamboo filler	233	282	381	395
18% bamboo filler	251	291	378	403
With residue of LW	249	288	392	400

**Table 8:** Weight loss data of polyurethane foams with rice husk filler.

Sample	5% Weight Loss	10% Weight Loss	Max DTG Point	50% Weight Loss
No filler	237	276	390	395
7% Rice husk filler	236	285	390	403
13% Rice husk filler	251	291	391	409
18% Rice husk filler	261	298	394	419

## Data Availability

The original contributions presented in the study are included in the article, further inquiries can be directed to the corresponding author.
